# Behavioral Plasticity in Ant Queens: Environmental Manipulation Induces Aggression among Normally Peaceful Queens in the Socially Polymorphic Ant *Leptothorax acervorum*

**DOI:** 10.1371/journal.pone.0095153

**Published:** 2014-04-17

**Authors:** Jürgen Trettin, Thomas Seyferth, Jürgen Heinze

**Affiliations:** Biologie I, Universität Regensburg, Regensburg, Germany; Universidade de São Paulo, Faculdade de Filosofia Ciências e Letras de Ribeirão Preto, Brazil

## Abstract

The behavioral traits that shape the structure of animal societies vary considerably among species but appear to be less flexible within species or at least within populations. Populations of the ant *Leptothorax acervorum* differ in how queens interact with other queens. Nestmate queens from extended, homogeneous habitats tolerate each other and contribute quite equally to the offspring of the colony (polygyny: low reproductive skew). In contrast, nestmate queens from patchy habitats establish social hierarchies by biting and antennal boxing, and eventually only the top-ranking queen of the colony lays eggs (functional monogyny: high reproductive skew). Here we investigate whether queen-queen behavior is fixed within populations or whether aggression and high skew can be elicited by manipulation of socio-environmental factors in colonies from low skew populations. An increase of queen/worker ratio and to a lesser extent food limitation elicited queen-queen antagonism in polygynous colonies from Nürnberger Reichswald similar to that underlying social and reproductive hierarchies in high-skew populations from Spain, Japan, and Alaska. In manipulated colonies, queens differed more in ovarian status than in control colonies. This indicates that queens are in principle capable of adapting the magnitude of reproductive skew to environmental changes in behavioral rather than evolutionary time.

## Introduction

The organization of animal societies and the underlying behavioral traits vary considerably among species but appear to be remarkably robust within species or at least within populations (e.g., [1]–[3]). For example, the share of individual group members in the offspring produced by the group (“reproductive skew”) ranges from equal partitioning among mutually tolerant group members (“low skew”) to reproduction being the privilege of only one individual, which dominates all other individuals (“high skew”; [4]–[6]). Reproductive skew may be affected by ecological constraints on solitary nesting, the relatedness and relative fighting strength of group members, and other factors (see e. g., [4], [7]–[9] and references therein). Consequently, reproductive skew and the causative behavioral processes typically vary less within than between species or populations [10]–[11].

In most social insects, queens in mature multi-queen societies show little antagonism and contribute equally to the offspring of the group (“polygyny”). High or even maximal skew has been described only from a small number of species with multi-queen colonies, where only one of several inseminated queens monopolizes reproduction ([12]–[15]). This “functional monogyny” [13] results from the formation of social rank orders among potential reproductives through overtly aggressive or ritualized dominance behavior (e.g., [12], [14]–[17]). The magnitude of reproductive skew and/or the associated behavior of queens in mature or founding colonies appear to be largely fixed within species [13] or at least within populations [18]–[21]. Whether queens are capable of adaptively reacting to changed environmental conditions, as assumed by models of optimal skew [22], has rarely been investigated.

Here, we examine whether experimental manipulation of colonies from a population with mutually tolerant queens and low skew can elicit queen-queen fighting similar to that underlying reproductive hierarchies in high-skew populations, i.e., whether queens are in principle capable of adjusting their reproductive behavior to changed environmental conditions. Our study species, the Holarctic ant *Leptothorax acervorum* (Fabricius, 1793), is socially polymorphic and the magnitude of reproductive skew varies among populations [18]–[19]. Sexuals of *L. acervorum* mate in late summer and thereafter young, mated queens may seek re-adoption into their natal nests. In low-skew populations, several nestmate queens continue to co-exist peacefully and lay eggs [18], [23]–[26]. In contrast, queens in high-skew populations engage in aggressive interactions and form dominance hierarchies after queen re-adoption and again after hibernation. Subordinate queens may stay in the nest as hopeful reproductives, leave the nest alone or together with workers from the natal nest to start a new colony, or are expelled through attacks by dominant queens and/or the workers [27]–[31]. Low-skew populations are abundant in the extended coniferous forests of boreal and alpine Central and Northern Eurasia with a high density of available nest sites for solitary foundresses [13], [23]–[26]. In contrast, high skew populations with social and reproductive rank orders live in quickly saturated habitat patches with nest site limitation [19], [27]–[31]. The distributional pattern of social organization parallels predictions from optimal skew models in that high skew appears to be associated with ecological constraints on solitary founding [32].

We aimed to elicit the formation of rank orders and high reproductive skew in colonies from a low-skew population by deteriorating the environmental conditions for queens. To do so, we experimentally increased queen-worker ratios in natural colonies and/or limited the availability of food. These treatments were motivated by studies according to which queen-worker ratios are considerably higher in functionally monogynous than polygynous species of *Leptothorax* [33] and food shortage may lead to increased skew in both ants [34]-[35] and social spiders [36]. Assuming that queen behavior and reproductive skew are plastic traits, we expected both manipulations to provoke queen-queen aggression and to lead to more pronounced differences in ovarian development. We show that in particular the reduction of worker number leads to queen fighting and more strongly skewed ovarian development.

## Materials and Methods

### Ant Collection and Rearing

Colonies of *L. acervorum* were collected from their nests in rotting branches in the well-studied low-skew population in Nürnberger Reichswald (June and August 2011), a pine-dominated forest near Nuremberg, Southern Germany (49°16′N, 11°10′E). Whole colonies were extracted from their nests in rotting twigs and transferred into standard three-chambered plastic boxes (10 cm×10 cm×3 cm) with plaster floor and reared under artificial spring/autumn conditions (12 h/12 h 20°C/10°C) in incubators as previously described [28], [37].

### Experimental Set-up

From freshly collected colonies we set up experimental colonies with 40 workers, 30 brood items and three to a maximum of seven dealate (wingless) queens found in the respective field colony. Queens were marked individually with 30 µm thin wires (copper or red enameled) tied between alitrunk and petiole, petiole and postpetiole, or postpetiole and gaster. Within three days after the set up, we subjected the experimental colonies to the following four treatments (nine colonies per treatment): control colonies without stressor, food-stressed colonies, severely worker-reduced colonies from which 20 workers were removed at the start of the experiment, and colonies which received a combined treatment of food- and worker-reduction. Colonies were assigned to the four treatment groups so that the averages and the distributions of initial queen numbers did not differ significantly among treatment groups (total initial queen numbers per treatment, control  = 44, food-stressed  = 49, worker-reduced  = 45, food- and worker-reduced  = 45; Kruskal-Wallis test, *H*_3_ = 1.331, *p* = 0.727; Kolmogorov-Smirnov two-sample tests, all *D*<0.444, all *p*>0.336).

Control and worker-reduced colonies were fed three-times during the observation period with chopped cockroaches and diluted honey ad libitum, food-stressed colonies only once. In the field, colonies often experience long periods during which workers cannot forage because of bad weather. Our food limitation experiment did not provide a similarly drastic reduction and colonies can well survive under this condition, albeit without investing a lot of resources into new brood. An observation time of 10 days was chosen as a trade-off, to keep the stressful period as short as possible and, at the same time, to guarantee the minimal sample sizes required to yield statistically meaningful results.

To keep queen-worker ratios high, we removed dark pupae and callow workers from the worker-reduced colonies (with and without food stress) on the fifth day of the observation period. This additional manipulation did not affect the behavior of queens and the frequency of aggression was similar on day five and six in the worker-reduced treatments (Wilcoxon signed rank test, W: *V* = 3, *n*_1_ = *n*_2_ = 8, *p* = 0.3711; FW: *V* = 3, *n*_1_ = *n*_2_ = 9, *p* = 1.0).

The experiment was carried out during two different observation periods (first round: five colonies per treatment, 2011-07-03 to 2011-07-12; second round: four colonies per treatment, 2011-08-29 to 2011-09-18. During this latter period not all experimental colonies could be simultaneously subjected to the various treatments).

### Observation and Ovary Dissection

Observations were started two days after the experimental manipulation. Each colony was observed under a binocular microscope in 20-min sessions twice per day over a period of ten consecutive days (total observation time per colony 400 min). Behavior was recorded by scan sampling every five minutes and in addition by ad libitum sampling [38]. The occurrence of all interactions involving queens (antennal boxing, mandible opening, biting, pulling, stinging/smearing, egg eating, egg laying, grooming, and trophallaxis, i.e., exchange of liquid food) was counted.

After the experiment, we killed all queens by freezing at −20°C and dissected their ovaries under a binocular microscope as described in [39]. We noted the presence of sperm in the spermatheca, corpora lutea, and mature oocytes and classified ovarian status as follows [16]: I undeveloped ovarioles, II slightly elongated ovarioles with a few immature oocytes, III elongated ovarioles, but no eggs in development (degenerated), IV fully elongated ovarioles with maturing oocytes, V fully elongated ovarioles with corpora lutea, but no eggs in development (degenerated). In addition, we recognized two intermediate stages (II–IV and IV–V).

Eighty-six percent of the observed queens (*n* = 155) were inseminated, the others were uninseminated and had shed their wings in the field without mating. Behavior of these virgin queens was excluded from the analysis because in *Leptothorax* they take over worker roles [40] and consequently do not represent an adequate substitute for mated queens. All colonies used in the analysis contained at least two mated queens except one worker-reduced colony, which was excluded from statistical analysis (for details on queen numbers per colony and treatment see Table S2). The exclusion of virgin queens or queens that died during the experiment led to slightly lowered average queen numbers relative to the beginning of the experiment, but as before neither average queen numbers nor the distribution of queen numbers were significantly different among the four treatments (total final queen number per treatment, control  = 38, food-stressed  = 45, worker-reduced  = 34, food- and worker-reduced  = 38; KW-test: *H*_3_ = 3.1086, *p* = 0.375; Kolmogorov-Smirnov two sample tests, all *D*<0.333, all *p*>0.699).

As was the aim of our manipulation, queen-worker ratios were significantly lower in control/food-stressed colonies than in both types of worker-reduced colonies (Mann-Whitney *U* test: *U* = 33.5, *n_1_* = 18, *n_2_* = 17, *p*<0.001). Experimental queen-worker ratios in control and food-stressed colonies were well within the range of queen-worker ratios from natural colonies in the low-skew population, while queen-worker ratios in worker-reduced colonies were similar to those previously reported from high skew populations (see Figure S1 and Table S1; but see [19]). The occurrence of queen mortality and the fact that the reproductive status of queens could only be determined after the observations ultimately resulted in a marginal overlap in queen-worker ratios among treatments (see boxplots A – D in Figure S1).

### Data Analysis

Preliminary analyses in a subset of colonies showed that egg-laying rate, the frequency of grooming between queens and the rate of aggression and trophallaxis from workers towards queens were too rare to give meaningful results in statistical test. We therefore omitted these types of behavior from the final statistical analysis.

We analyzed the effect of the different treatments on the respective behavioral responses per colony by Scheirer-Ray-Hare tests (SRH), a non-parametric equivalent for a multi-way ANOVA [41], with worker reduction and food reduction as independent factors. To account for the two different observation periods we included “time” as a third factor. *P*-values were adjusted for multiple tests (queen-queen aggression, egg eating, trophallaxis, grooming) using sequential Bonferroni corrections [42]. In addition, we used Kruskal-Wallis tests to analyze data for an overall effect of different treatments on queen-queen aggression per colony for each observation period separately. To determine whether queens contribute equally to aggression we estimated the B-index [43]. For the statistical comparison of ovarian development among treatments we combined manipulated colonies due to the small sample size in each category.

All statistical analyses were carried out in R version 2.14.1 to 3.0.1 [44] or PAST v. 1.75b [45].

### Ethics statement

As no protected species was sampled and colonies were collected only from a state-owned forest, no permits and approval for ant collection were required. All experiments comply with national and international law.

## Results

### Queen-Queen and Worker-Queen Behavior

**Figure 1:**
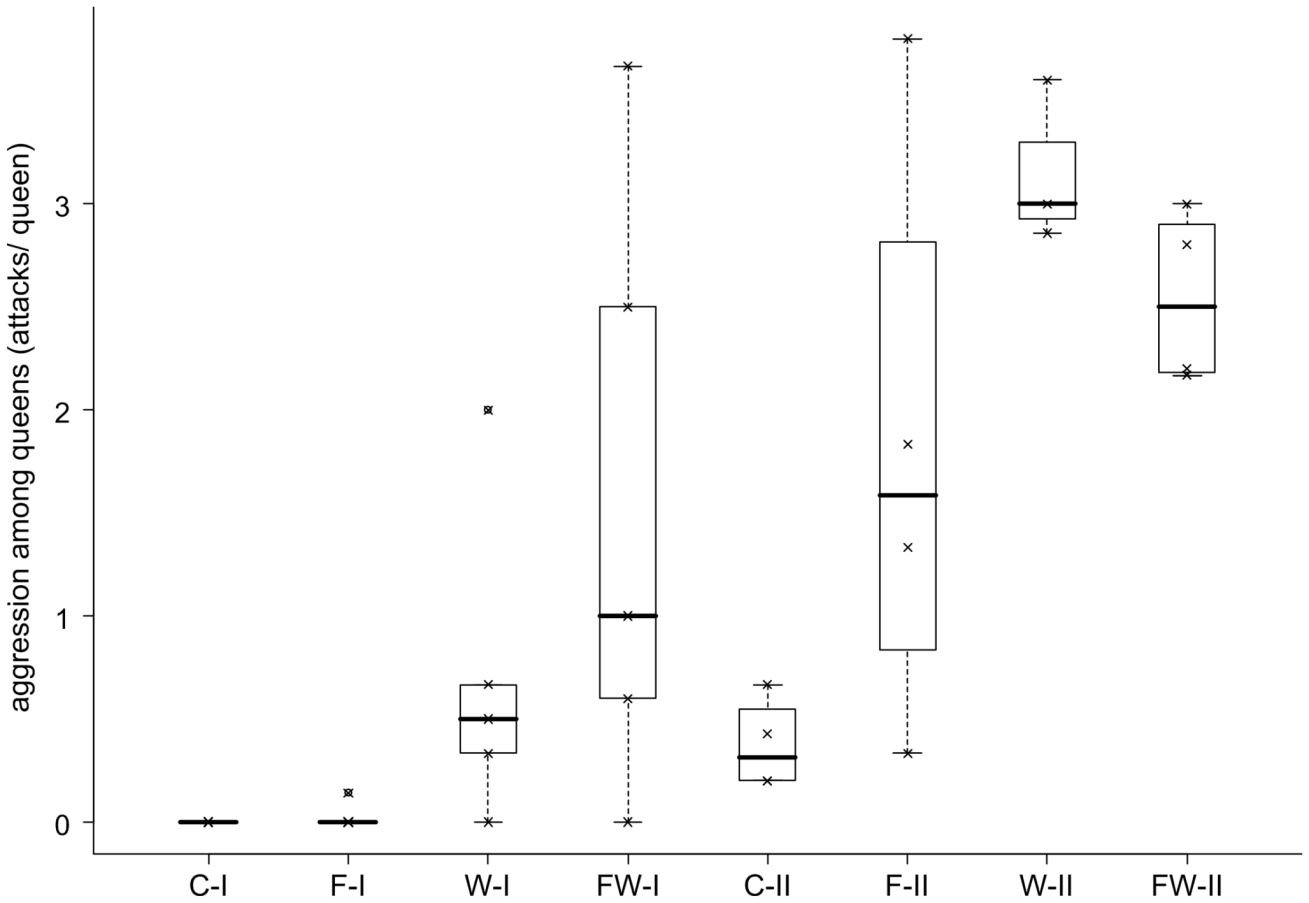
Frequency of aggression among queens of the ant *Leptothorax acervorum* from a low-skew population. Data shown as attacks per queen during the total observation period (median, quartiles, range). Individual colonies were subjected to different types of stress (food reduction F, worker reduction W, or both FW) or left unmanipulated (control C) in two different seasons (July, I, and September, II). Outliers are indicated as ◯ and original data points as ×.

Experimental manipulation of colonies from the low-skew Reichswald population resulted in queen-queen antagonism similar in quality and quantity to that previously observed in high-skew populations (antennal boxing, mandible opening, biting, pulling and stinging/smearing; [28], [30], [31]). In total, we observed 187 attacks among queens during 230 hours of direct observation (107 instances of antennal boxing, 21 threats with opened mandibles, 53 bites, 4 dragging on legs or antennae, 2 stings). The occurrence of aggressive behavior differed greatly among the four treatments and also between the observation periods (Figure 1). A Scheirer-Ray-Hare test gave evidence for a strong influence of the factors “observation period” (SRH: *H*_1_ = 12.68, *n* = 26, *p* = 0.0005; corrected for multiple tests, *p*′ = 0.0021) and “worker reduction” (SRH: *H*_1_ = 10.17, *p* = 0.0014, *p*′ = 0.0057). Averaged over all treatments, more aggression occurred during the second observation period (median, quartiles, first observation period: 0, 0, 0.617 attacks per queen, *n* = 20; second observation period: 2.167, 0.548, 2.928 attacks per queen, *n* = 15, Mann-Whitney *U* test: *U* = 50, *p*<0.0008). Statistical analysis also revealed significant differences between treatments for each observation period separately (Kruskal-Wallis tests, first observation period: *H*_3_ = 10.88, *p* = 0.01239; second observation period: *H*_3_ = 8.79, *p* = 0.0323).

In accordance with previous observations [18], queen-queen aggression almost never occurred in control colonies (median, quartiles 0, 0, 0.2 attacks per queen). The reduction of worker number alone and in combination with food reduction resulted in a considerable increase of aggressive behavior among queens (median, quartiles 2.1, 0.6, 2.9 attacks per queen). In principle, the drastically changed queen-worker ratio, the lower worker number and the experimental removal of workers and worker pupae might all have elicited queen-queen antagonism. However, the following observation suggests that queen aggression is a consequence of manipulated queen-worker ratio and not of disturbance or lower worker number alone: in one of the nine worker-reduced colonies, in which dissection at the end of the experiment revealed that several queens were not inseminated and queen-worker ratio thus was not greatly changed, no aggression was observed. Furthermore, over all worker-reduced colonies, the number of attacks appeared to increase with queen-worker ratio, albeit not significantly so (Gamma correlation: *Γ* = 0.353, *n* = 17, *p* = 0.083). Neither food-reduction nor worker-reduction had a significant effect on the level of aggression from workers towards queens (SRH, food-reduction: *H*_1_ = 1.30, *p* = 0.255; worker-reduction: *H*_1_ = 0.13, *p* = 0.717).

Food reduction alone did not have a significant effect on queen aggressiveness (SRH: *H*_1_ = 1.15, *p* = 0.283, *p*′ = 0.850). In none of the behaviors were interactions among the various factors significant (all *p*>0.07). The short duration of the experimental manipulation did not allow deducing rank orders from the aggression. Nevertheless, the contribution of individual queens to the aggression was significantly different from random in three of those eight colonies with worker reduction (and worker reduction plus food reduction), in which more than 10 aggressive interactions among queens were observed (B-indices of 0.304, 0.978, and 0.214, with the confidence intervals not overlapping 0). Worker reduction in addition led to an increase of egg eating by queens (SRH: *H*_1_ = 6.19, *n* = 26, *p* = 0.0129, *p*′ = 0.0350, Figure S2), and food reduction significantly increased the frequency of trophallaxis between queens (SRH: *H*_1_ = 9.31, *n* = 27, *p* = 0.0023, *p*′ = 0.0091, Figure S3). Queens from worker-reduced colonies were more frequently groomed by workers than queens from food-reduction colonies (SRH: *H*_1_ = 6.36, *n* = 26, *p* = 0.0117, *p*′ = 0.0350, Figure S4).

### Ovarian status

Ovarian status differed greatly between the observation periods. The ovaries of queens observed in September were elongated and contained corpora lutea, suggesting that the queens had been fully fertile, but they rarely contained maturing oocytes. Only six of 80 queens had still fully developed ovaries (stage IV), while the majority of queens had ovaries of stage V and probably had started to prepare for hibernation. We therefore did not examine differences in ovarian status among the different treatments in these colonies. In contrast, in July all five control colonies and nine of 14 stressed colonies (3 of 5 colonies with food reduction, 4 of 5 colonies with worker reduction and 2 of 4 colonies with both manipulations), contained one or several fully fertile queens with stage IV ovaries. Four of five control colonies but only two of 14 stressed colonies contained two or more fully fertile queens (Fisher's exact test, *p* = 0.017). Other queens had degenerated their ovaries.

## Discussion

We investigated whether queens from a low-skew population of the ant *Leptothorax acervorum* adjust their behavior towards nestmate queens and the partitioning of reproduction in response to experimentally changed conditions. Our data show that experimental manipulation, in particular the reduction of worker numbers, provoked fighting and dominance interactions similar in quality and quantity to those previously observed in high-skew populations of this and other *Leptothorax* species. Though the absolute number of queen-queen attacks was low, few and infrequent interactions may suffice to establish clear social and reproductive rank orders among ants (e.g., [31]). Food reduction alone did not lead to an increase in aggression but resulted in a higher frequency of food begging and food exchange among queens.

In high skew populations of *L. acervorum* and other functionally monogynous ants queen-queen aggression establishes social rank orders among queens [14], [16], [28], [30], [31]. The incidence of aggression peaks directly after hibernation when queens begin to mature eggs and again in fall when queens prepare for hibernation and young, adopted queens integrate themselves into the hierarchy [46]. This temporal fluctuation of aggressiveness might explain the large difference in the frequency of queen antagonism between the two observation periods also in our study. The rank order of queens in functionally monogynous ants determines their reproductive status, and usually only the top-ranking queen lays eggs.

Dissections of queens in our experiment indicated that already after ten days of manipulation, queens from worker- and food-stressed colonies differed more strongly in their ovarian status than queens from control colonies. In most stressed colonies, only one queen had remained fully fertile, while other queens had begun to degenerate their ovaries. We cannot exclude that our experimental manipulation directly affected ovarian status in some queens more than in others. However, comparison with natural high-skew colonies suggests that the observed variation results from aggression and/or an increased rate of food exchange from subordinate to dominant queens. Begging food from subordinates is a subtle mechanism of domination through which queens in related species deplete workers of the resources needed to produce eggs [47], and food stress was reported to increase reproductive skew in the ant *Myrmica rubra* [34]. In our study, worker reduction also led to an increased frequency of egg-eating by queens. Egg eating by *L. acervorum* queens is well documented, and queens in low skew populations apparently do not discriminate between their own eggs and those laid by other queens [23], [48]. Such indiscriminate oophagy does not alter stable skew. In contrast, Ito [30] observed that dominant queens from a high-skew population on Hokkaido fed on eggs immediately after these were laid by subordinate queens and in this way increased reproductive skew. Unfortunately, we did not determine the origin of eggs in our study, but it is possible that egg eating would have increased skew in stressed colonies.

Ideally we should also have performed the opposite experiment, i.e., trying to induce queen tolerance in colonies from high skew populations of *L. acervorum* by adding workers or overfeeding the colonies. However, documenting the complete disappearance of a rare behavioral trait is more difficult than documenting its induction. Furthermore, queen aggressiveness and reproductive skew appear to vary to some extent among natural colonies of high skew populations of *L. acervorum* and related species, and not in all studied colonies were queens seen to engage in aggressive interactions [19], [28], [31], [49].

Our result that an increase of the queen-worker ratio increases queen-queen antagonism and reproductive skew matches the observation that queen-worker ratio is higher in high-skew species than in low-skew species [33]. Queen-worker ratio might co-vary with ecological constraints on solitary colony founding that have been suggested to affect the magnitude of reproductive skew. A high queen-worker ratio can result from a high re-adoption rate of young queens or high mortality of workers, e. g., during hibernation. Both might reflect adverse environmental conditions, which pose high costs on dispersal and colony founding by solitary queens [32]. In addition, the chance for individual queens to found new colonies accompanied by workers might be more limited at higher queen-worker ratios.

The easy induction of queen-queen aggression and increased reproductive skew in colonies in which queens normally do not fight is at odds with results from previous studies on social plasticity in ants. For example, the social structure of colonies of the red imported fire ant, *Solenopsis invicta*, depends on the genotypes of queens and workers at a particular locus [50], and whether co-founding queens of *Messor pergandei* and *Pogonomyrmex californicus* exhibited aggression or were mutually tolerant did not depend on group size but on region of origin [20], [21]. Similarly, queens of *Temnothorax rugatulus* were not capable of expanding their behavior to that shown during the workerless colony founding phase under experimental worker shortage [51]. Our finding also contrasts with previous studies in *L. acervorum*: dissections and population genetic analyses suggest that the level of skew is more or less fixed within populations, and that colonies maintain their social phenotype when kept under standardized laboratory conditions [18], [19], [25], [26]. Therefore, different levels of reproductive skew have been suggested to be an evolved rather than a behavioral response [19], [49]. This discrepancy might mean that ants from different populations have evolved different thresholds for fighting and dominance behavior, leading to more or less fixed local social organization. In addition, variation in skew within populations may have been underestimated by genetic analyses and dissections. Skew estimates from the genetic assignment of maternity of adults are often difficult because of the typically high relatedness among nestmate queens and rapid queen turnover. Genotyping of eggs and dissections give only a snap-shot account of the status quo of the partitioning of brood production. As it may be difficult to distinguish between formerly active, but now degenerate ovaries and not yet activated ovaries, the past or future contributions of queens that presently do not mature eggs may be underrated.

### Conclusions

Our study demonstrates that queens of *L. acervorum* from low skew populations are able to react to changes in their social environment. In addition, it highlights the importance of queen-worker ratio for the adjustment of skew and the need for further studies to clarify its role and the role of other factors (e. g., egg cannibalism and habitat structure) in the formation and maintenance of reproductive skew in insect societies.

## Supporting Information

Figure S1 Queen-worker ratios of experimental treatments from this study (grey) as well as natural queen-worker ratios from several low skew (red) and high skew (blue) populations. (A: controls, B: food reduction, C: worker reduction, D: both treatments (FW), E–K: correspond to references in Table S1; E = 1, F = 2, G & H & I = 3, J = 4, K = 5 & 6).
(TIF)

Figure S2 Frequency of egg eating (observations per queen during the total observation period, median, quartiles, range) in colonies of the ant *Leptothorax acervorum* from the low-skew population in Nürnberger Reichswald. Individual colonies were subjected to different types of stress (food reduction F, worker reduction W, or both FW) or left unmanipulated (control C) in two different seasons (July, I, and September, II). Outliers are indicated as circles.
(TIF)

Figure S3 Frequency of trophallaxis, i.e., food exchange, among queens (observations per queen during the total observation period, median, quartiles, range) of the ant *Leptothorax acervorum* from the low-skew population in Nürnberger Reichswald. Individual colonies were subjected to different types of stress (food reduction F, worker reduction W, or both FW) or left unmanipulated (control C) in two different seasons (July, I, and September, II). Outliers are indicated as circles.
(TIF)

Figure S4 Frequency of queens groomed by workers (observations per queen and worker during the total observation period, median, quartiles, range) of the ant *Leptothorax acervorum* from the low-skew population in Nürnberger Reichswald. Individual colonies were subjected to different types of stress (food reduction F, worker reduction W, or both FW) or left unmanipulated (control C) in two different seasons (July, I, and September, II). Outliers are indicated as circles.
(TIF)

Table S1 Queen-worker ratios in high skew and low skew populations of the ant *Leptothorax acervorum*.
(DOCX)

Table S2 Number of inseminated queens and initial queens (in brackets) per replicate colony and treatment. Individual colonies were subjected to different types of stress (food reduction F, worker reduction W, or both FW) or left unmanipulated (control C).
(DOCX)
