# Supplementary figures and images for: Behavioral Plasticity in Ant Queens: Environmental Manipulation Induces Aggression among Normally Peaceful Queens in the Socially Polymorphic Ant Leptothorax acervorum
Source: PLoS One. 2014 Apr 17;9(4):e95153. doi: 10.1371/journal.pone.0095153 (PMC3990625; doi:10.1371/journal.pone.0095153)

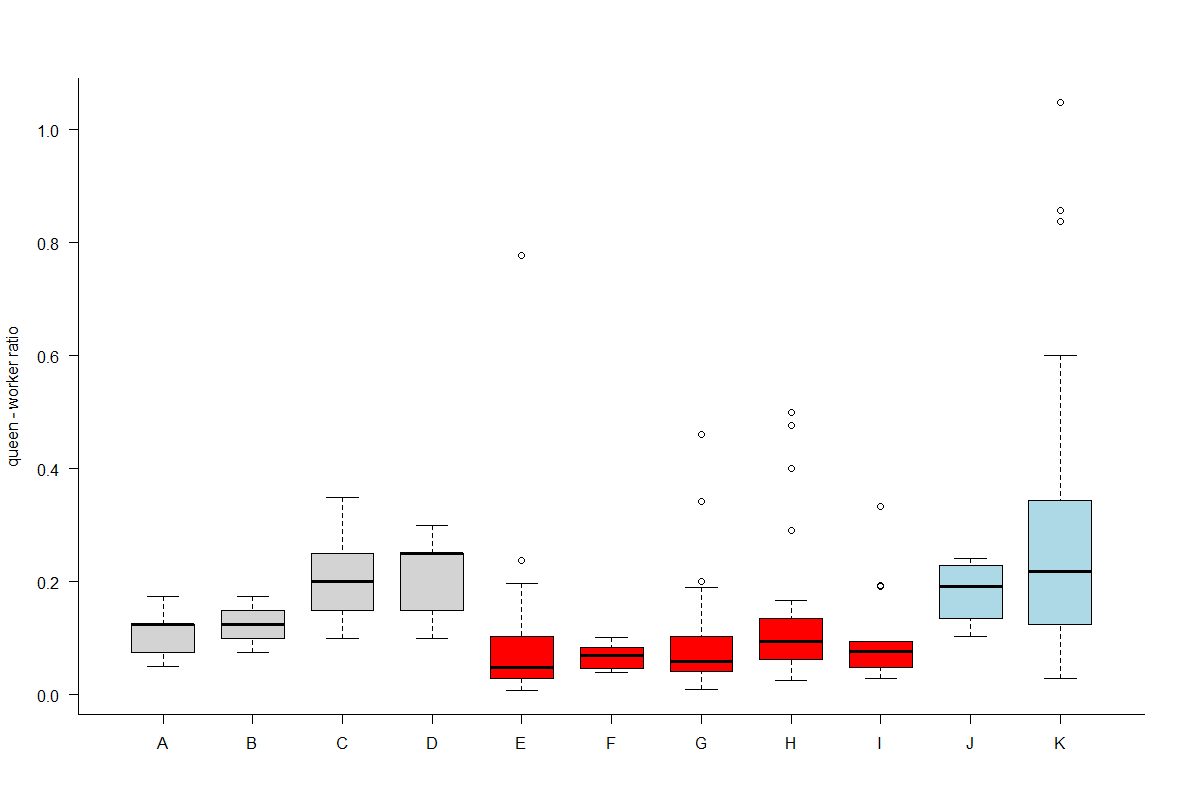

Supplement: Figure S1 — Queen-worker ratios of experimental treatments from this study (grey) as well as natural queen-worker ratios from several low skew (red) and high skew (blue) populations. (A: controls, B: food reduction, C: worker reduction, D: both treatments (FW), E–K: correspond to references in Table S1; E = 1, F = 2, G & H & I = 3, J = 4, K = 5 & 6). (TIF) [file pone.0095153.s001.tif]
