# Supplementary material for: Behavioral Plasticity in Ant Queens: Environmental Manipulation Induces Aggression among Normally Peaceful Queens in the Socially Polymorphic Ant Leptothorax acervorum
Source: PLoS One. 2014 Apr 17;9(4):e95153. doi: 10.1371/journal.pone.0095153 (PMC3990625; doi:10.1371/journal.pone.0095153)
Supplement: Table S1 — Queen-worker ratios in high skew and low skew populations of the ant Leptothorax acervorum. (DOCX) [file pone.0095153.s005.docx]

**Table S1.** Median queen – worker ratios for low-skew and high-skew populations of *Leptothorax acervorum*.

| population | # colonies | queens/ colony | workers/ colony | queen – worker ratio | social structure | reference |
| --- | --- | --- | --- | --- | --- | --- |
|  | (*n*)**^¶^** |  |  |  | reproductive skew |  |
| Germany | 35 | 3.0 (2.5, 5.5) | 86.0 (57.0, 120.0) | 0.049 (0.030, 0.103) | facultative polygyny  low | [1] |
| UK | 5 | 8.0 (7.0, 11.0) | 127.0 (95.0, 151.0) | 0.070 (0.046, 0.084) | facultative polygyny  low | [2] |
| UK (Santon) * | 31 | 4.0 (3.0, 5.5) | 62.0 (42.0, 121.5) | 0.059 (0.041, 0.103) | facultative polygyny  low | [3] |
| UK (Roydon) | 25 | 3.0 (2.0, 7.0) | 48.0 (25.0, 68.0) | 0.095 (0.063, 0.135 | facultative polygyny  low | [3] |
| UK (Aberfoyle) * | 13 | 10.0 (5.0, 20.0) | 174.0 (68.0, 514.0) | 0.077 (0.049, 0.095) | facultative polygyny  low | [3] |
| Japan | 4 | 5.5 (5.0, 6.3) | 32.5 (27.5, 39.0) | 0.192 (0.151, 0.223) | functional monogyny  high | [4] |
| Spain * | 50 | 14.5 (6.0, 21.8) | 54.5 (32.0, 77.3) | 0.219 (0.125, 0.337) | functional monogyny  high | [5, 6] |

Quartiles are given in parenthesis. * Populations were sampled during two different seasons. **^¶^** Only colonies with two or more queens (polygynous) were used in the analysis.

**References**

[1] Heinze J, Lipski N, Schlehmeyer K, Hölldobler B (1995) Colony structure and reproduction in the ant *Leptothorax acervorum*. Behav Ecol 6: 359-367.

[2] Bourke AFG (1991) Queen behaviour, reproduction and egg cannibalism in multiple- queen colonies of the ant *Leptothorax acervorum*. Anim Behav 42: 295-310.

[3] Chan GL, Hingle A, Bourke AFG (1999) Sex allocation in a facultatively polygynous ant: between-population and between-colony variation. Behav Ecol 10: 409-421.

[4] Ito F (2005) Mechanisms regulating functional monogyny in a Japanese population of *Leptothorax acervorum* (Hymenoptera, Formicidae): dominance hierarchy and preferential egg cannibalism. Belg J Zool 135: 3-8.

[5] Felke M, Buschinger A (1999) Social organization, reproductive behaviour and ecology of *Leptothorax acervorum* (Hymenoptera, Formicidae) from the Sierra de Albarracin in central Spain. Insectes Soc 46: 84-91.

[6] Felke M (1999) Biologie, soziogenetische Kolonie- und Populationsstruktur, sowie taxonomische Stellung einer zentralspanischen *Leptothorax acervorum*-Population (Hymenoptera; Formicidae). Doctoral thesis, Technische Universität Darmstadt.
