# Supplementary material for: Behavioral Plasticity in Ant Queens: Environmental Manipulation Induces Aggression among Normally Peaceful Queens in the Socially Polymorphic Ant Leptothorax acervorum
Source: PLoS One. 2014 Apr 17;9(4):e95153. doi: 10.1371/journal.pone.0095153 (PMC3990625; doi:10.1371/journal.pone.0095153)
Supplement: Table S2 — Number of inseminated queens and initial queens (in brackets) per replicate colony and treatment. Individual colonies were subjected to different types of stress (food reduction F, worker reduction W, or both FW) or left unmanipulated (control C). (DOCX) [file pone.0095153.s006.docx]

**Table S2.** Number of initial (in brackets) and inseminated queens per replicate colony and treatment.

| replicate | treatment |  |  |  |
| --- | --- | --- | --- | --- |
| colonies | C | F | W | FW |
| I | 5 (6) | 5 (6) | 3 (3) | 3 (3) |
| II | 2 (3) | 4 (4) | 5 (6) | 5 (6) |
| III | 5 (5) | 3 (3) | 3 (4) | 3 (3) |
| IV | 3 (4) | 3 (6) | 2 (3) | 4 (5) |
| V | 3 (4) | 7 (7) | 3 (4) | 2 (5) |
| VI | 3 (3) | 6 (6) | 5 (6) | 5 (5) |
| VII | 5 (6) | 5 (5) | 1 (5)* | 5 (7) |
| VIII | 7 (7) | 6 (6) | 5 (7) | 6 (6) |
| IX | 5 (5) | 6 (6) | 7 (7) | 5 (5) |

* Colony was removed from analysis.
